# Psychotraumatology on the move

**DOI:** 10.1080/20008198.2018.1439650

**Published:** 2018-03-06

**Authors:** Miranda Olff

**Affiliations:** ^a^ Editor-in-Chief European Journal of Psychotraumatology, Department of Psychiatry, Academic Medical Center, University of Amsterdam, & Arq Psychotrauma Expert Group, Diemen, The Netherlands

**Keywords:** psychotrauma, PTSD, Open Access, impact factor, altmetrics, special issues, psicotrauma, trastorno de estrés postraumático (TEPT), acceso abierto, factor de impacto, Altmetrics, problemas especiales, 心理创伤, PTSD, 开放获取, 影响因子, 替代计量学, 特刊, • More and more research is published Open Access, especially in psychotraumatology.• *European Journal of Psychotraumatology* (EJPT) is Open Access, well read and cited, and has a global impact.

## Abstract

Psychotraumatology is on the move. Worldwide, there is an increasing awareness of the negative impact of psychotrauma, which is reflected in the number of publications on the topic. These publications become more and more available to the public (Open Access), even more so than in other fields, and thus lead to quicker implementation of research findings. In this editorial, the editor of the *European Journal of Psychotraumatology* (EJPT) provides a brief review of how the journal has been doing, including recent accomplishments, some metrics and the ESTSS EJPT award for best paper of 2017, and identifies a number of priorities for the next year.

## Abstract (es)

La psicotraumatología está avanzando. En todo el mundo existe conciencia cada vez mayor conciencia del impacto negativo del psicotrauma, que se refleja en la cantidad de publicaciones sobre el tema. Estas publicaciones están cada vez más disponibles para el público (acceso abierto), incluso más que en otros campos, y por lo tanto dan lugar a una implementación más rápida de los resultados de la investigación. En este editorial, el editor del *European Journal of Psychotraumatology* (*Revista europea de psicotraumatología*, EJPT) proporciona una breve reseña de cómo ha estado la revista e incluye los logros recientes, algunas mediciones, el premio ESTSS EJPT al mejor artículo de 2017 e identifica una serie de prioridades para el el próximo año.

## Abstract (zh)

心理创伤学正在前行。从相关主题的发表文章的数量上可以看出，世界范围内已经越来越多认识到心理创伤的负面影响。这些文章变得越来越易得（开放获取），甚至超过其它的领域。因此也使得将研究发现投入实践变得更快了。在这篇编辑评论中，《欧洲心理创伤学期刊》的主编简要概括了本刊的情况，内容包括了最近的成就、一些图表，和 ’2017 ESTSS EJPT 最佳论文奖’结果，并对下一年的优先计划作出了总结。

## 1. Psychotraumatology on the move

**Figure 1.**
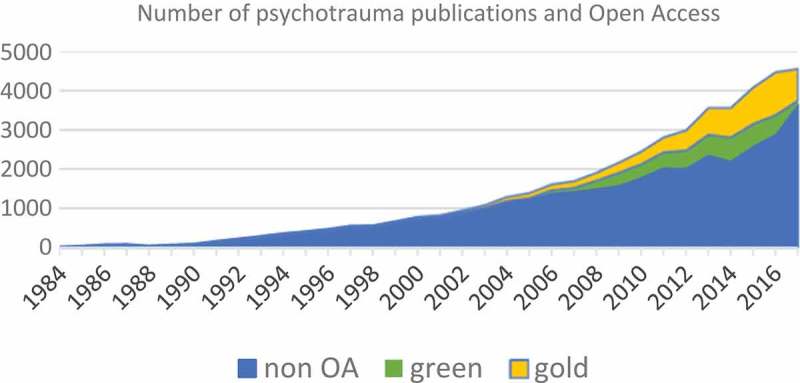
Number of psychotrauma related publications and Open Access. Web of Science 23 January 2018. Indexes: SCI-EXPANDED, SSCI, A&HCI, ESCI. Publications years 1984–2017. **TOPIC**: (PTSD) *OR* **TOPIC**: (post-traumatic stress) *OR* **TOPIC**: (psychotrauma) *OR* **TOPIC**: (psychotraumatology) = 47,026 records. Analysis: **Open Access**: (GOLD): 6,786 records (GREEN ACCEPTED OR GREEN PUBLISHED): 4,391 records.

Worldwide, there is an increasing awareness of the impact of psychotrauma (e.g. Magruder, McLaughlin, & Elmore Borbon, 2017) with an associated increase in the number of papers published on psychotrauma (Figure 1). There is also an increasing number of journals that now focus on psychotrauma; of those, only one is fully Open Access (Gold), and that is *European Journal of Psychotraumatology* (EJPT), owned by the European Society of Traumatic Stress Studies (ESTSS). With our *Gold Open Access* model, we reach researchers, practitioners and policy-makers, around the world, who otherwise may not have had access to this information.

**Table 1.** ESTSS EJPT best paper of 2017 top 5.

1.	Does size really matter? A multisite study assessing the latent structure of the proposed ICD-11 and DSM-5 diagnostic criteria for PTSD – Hansen et al. (2017)
2.	Trauma and PTSD in the WHO World Mental Health Surveys – Kessler et al. (2017)
3.	Neuroimaging the traumatized self: fMRI reveals altered response in cortical midline structures and occipital cortex during visual and verbal self- and other-referential processing in women with PTSD – Frewen et al. (2017)
3.	Tetris and Word games lead to fewer intrusive memories when applied several days after analogue trauma – Hagenaars et al. (2017)
3.	The Neural correlates of childhood maltreatment and the ability to understand mental states of others – C. Van Schie et al. (2017)

In general, we can say that the journal is doing well. Papers are actually being downloaded and cited (see below), resulting in an increasing impact factor, but most importantly reaching readers all around the globe. The messages from practitioners from all corners of the world –including Western developed countries – expressing their appreciation for easily having access to EJPT articles and on our open mind to address different areas of the field, demonstrate that we are doing the right thing.

The Open Access policy is slowly but steadily becoming accepted as the way forward in sharing academic data. More and more funders and institutes support Open Access, or even require making research funded with public money available to the larger public [e.g. European Commision (H2020), Welcome Trust, National Institutes of Health, The Netherlands Organization for Scientific Research (NWO)], and researchers realize that their work is better read, used and cited when having it freely available without barriers to access.

*Gold Open Access* means that researchers publish their articles in fully Open Access online academic journals that do not have subscriptions and are therefore accessible to everybody. NWO (like other funders) prefer *Gold Open Access* (see www.nwo.nl). In the case of *Green Open Access*, researchers publish their articles in a traditional subscription journal, but a version of the article is also made accessible via a public database (e.g. a university repository) at the same time as this publication. There is also the ‘hybrid’ Open Access form where researchers publish in a traditional subscription journal but pay Article Processing Charges (APCs) to make this article freely accessible.

The number of Open Access publications over the past decade is sharply increasing, including in the psychotrauma area (Figure 1). In 2017, 17.7% of psychotrauma research was *Gold Open Access*, while this is 14.6% in psychology and psychiatry journals together and 16.8% in all journals indexed in Web of Science in 2017. The expectation is that this trend will continue to grow exponentially.

**Figure 2.**
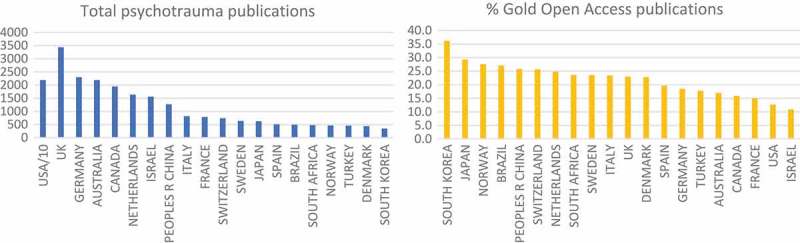
Number of psychotrauma related publications and percentage Gold Open Access by country. Web of Science 23 January 2018. Idem. Refined by: Open Access: (ALL OPEN ACCESS) AND Open Access: (GOLD). Indexes: SCI-EXPANDED, SSCI, A&HCI, ESCI. Timespan: All years. **TOPIC**: (PTSD) *OR* **TOPIC**: (post-traumatic stress) *OR* **TOPIC**: (psychotrauma) *OR* **TOPIC**: (psychotraumatology).

In absolute terms, the U.S., U.K., Germany, Australia and Canada publish the largest number of psychotrauma-related papers (according to Web of Science); however the percentage of these papers that is Open Access is highest in South Korea, Japan, Norway, Brazil and China, likely reflecting the strong Open Access and more general Open Science policies in these countries (Figure 2).

In December 2017, EJPT celebrated its 7th birthday. To mark this birthday, a collection of the best articles from the last few years has been created. These papers have a wide range of scope covering psychotherapy to neurobiology, trials to reviews, and all of these can be found here. We have great plans for 2018 (see below) to continue publishing papers on how to understand, prevent and treat the consequences of stress and trauma including post-traumatic stress disorder (PTSD), depressive disorders, substance abuse, burnout and neurobiological, physical or any other consequences, in animals or humans, children or adults. Below, we present our accomplishments in 2017 and look forward to 2018.

## 2. 2017 special issues

### 2.1. Populations and methods on the move

We publish special issues on topics that are timely or deserve extra attention and for which we often invite a guest editor who has expertise in the specific area. Please note that when the call is closed, it does not mean that we are no longer interested in the topic; papers can always be added to the issue at a later point in time. Please check out the special issues page for more detail.

#### 2.1.1. Traumatized refugees

Last year, we focused on traumatized refugees: identifying needs and challenges faced for mental health care, guest-edited by Christine Knaevelsrud and Nadine Stammel (Knaevelsrud, Stammel, & Olff, 2017). These guest editors did a fantastic job in bringing together three review articles and four original articles on the mental health burden, screening instruments and interventions in different groups of refugees. With the number of refugees worldwide increasing dramatically in the past few years, we hope this issue will help us better understanding their needs and will improve the provision of appropriate mental health care for this population.

#### 2.1.2. Bayesian statistics

The idea of making optimal use of existing information as in Bayesian statistics is an attractive way forward in psychotraumatology research. We therefore published a special issue containing six papers on the use of Bayesian statistics (van de Schoot, Schalken, & Olff, 2017) including an introduction on how and why Bayesian statistics are being applied, e.g. for flexible hypothesis testing, updating probabilities, no need for large data sets, imputation of missing data and allowing authors to address technical complexities.

#### 2.1.3. PTSD symptomics

The third special issue on PTSD symptomics guest-edited by Cherie Armour and Eiko Fried involves research on network structures of PTSD symptoms (Armour, Fried, & Olff, 2017). We believe that the network approach to psychopathology has been embraced so quickly ‘because it reflects how patients and clinicians think about many mental disorders: as dynamic systems of causal influences and vicious circles […] in contrast with the frameworks adopted by the DSM and ICD that understand symptoms as passive consequences of underlying disorders’ (Armour et al., 2017). We are looking forward to seeing whether this exciting new approach can lead to significant changes in how mental disorders are measured, modelled, diagnosed and eventually treated.

#### 2.1.4. Conference abstracts

We also published the abstracts of the first psychotrauma meeting in Athens (see Kolaitis & Olff, 2017). Abstracts are not being peer-reviewed except by the scientific committee of the conference, and the findings in the full papers may differ from those in abstracts (see Li et al., 2017). However, this is an efficient way to share the latest research developments with the wider world as presented at conferences, and to stay updated on where psychotraumatology in different areas of the world is moving. Please contact the editor if you would like to share your conference abstracts.

## 3. ESTSS EJPT best paper of 2017 award

Associate and guest editors, members of the editorial board, abstract translators and members of the ESTSS board were invited to nominate papers for the *ESTSS EJPT 2017 award*. Criteria were free and broad, for example: sound methodology, relevance for clinical practice, innovation, global reach, outstanding science, theoretically advancing the field, thought provoking, etc. We received 56 nominations. Interestingly, but not surprisingly considering the criteria, they were quite diverse, and also the reasons given for why this paper would deserve the award reflected all criteria mentioned above. The top 5 is listed in Table 1.

We have a winner: the ESTSS EJPT 2017 award goes to *Maj Hansen* and colleagues for ‘Does size really matter? A multisite study assessing the latent structure of the proposed ICD-11 and DSM-5 diagnostic criteria for PTSD’ (Hansen et al., 2017). And this is not for the title that some of us found ‘intriguing’ but for its relevant and timely content, the importance of the comparison of two diagnostic systems and the use of three large samples (*N* = 4,213), the diversity of the samples (clinical, work related and community), the use of validated tools to measure both the ICD-11 and the DSM-5 PTSD symptoms, and the fact that both quantitative and qualitative differences in prevalence rates were investigated. Some of the arguments given included: ‘This is an important large-sample study comparing the two diagnostic systems DSM-5 vs. ICD-11 regarding factor structure and prevalence rates. I think it is timely and clinically significant. Results tend to be in favour of the simpler ICD system.’ ‘… and the study allows to make particular conclusions which are useful in both theoretical and practical sense’.

Interestingly, number 2 on the list also included an impressive number of subjects: ‘Trauma and PTSD in the WHO World Mental Health Surveys’ by Kessler et al. (2017). ‘The study assessed 29 lifetime trauma in 68,894 persons from 24 countries, a wonderful summary of cross-national findings, and it highlights the adverse effects of intimate partner violence.’

Three papers shared places 3 to 5. The study by Frewen, Thornley, Rabellino, and Lanius (2017) ‘Neuroimaging the traumatized self: fMRI reveals altered response in cortical midline structures and occipital cortex during visual and verbal self- and other-referential processing in women with PTSD’ because it ‘is the first to compare valenced self vs other-referential processing in persons with PTSD using fMRI. The findings may provide bases for neuroscience-informed treatments.’

The next study is: ‘Tetris and Word games lead to fewer intrusive memories when applied several days after analogue trauma’ (Hagenaars, Holmes, Klaassen, & Elzinga, 2017) because ‘it represents an effort to succinctly evaluate important novel theories in order for us to learn more about how to address specific trauma-related symptoms’.

Finally, ‘The Neural correlates of childhood maltreatment and the ability to understand mental states of others’ by C. Van Schie et al. (2017) study ‘combines fMRI and the assessment of the ability to understand mental states of others which is a very important living ability, in adolescents with childhood maltreatment …’ or ‘.. for its scientific innovation, sophisticated formulation of the problems associated with childhood maltreatment and potential impact on theory and clinical practice.’

**Figure 3.**
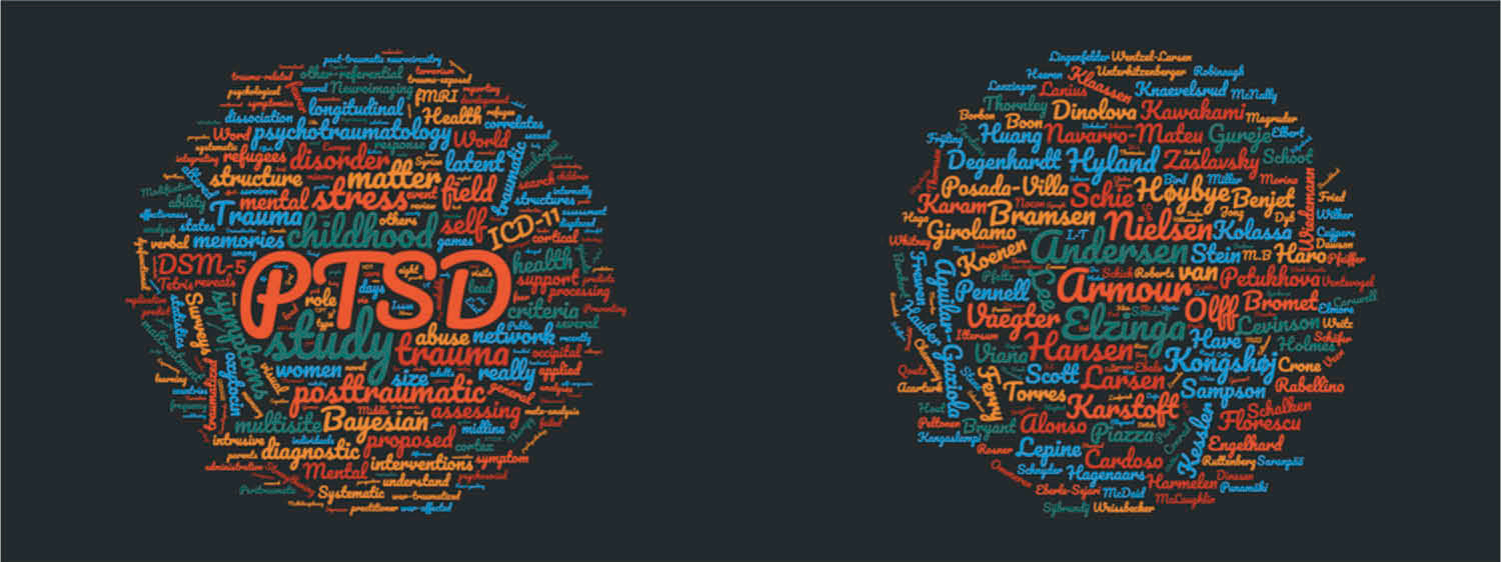
Word clouds of titles and author names of all nominated papers for the ESTSS EJPT 2017 award. Created with https://www.wordclouds.com/. Larger size indicates more votes.

I am pleased to see how people like the variety of papers (see also Figure 3), and a few papers that did not quite make it into the top 5 do reflect this diversity, e.g. neurobiological research, like the oxytocin paper by Frijling (2017), the methodological ones, e.g. McNally, Heeren, and Robinaugh (2017)’s paper on Bayesian statistics, and public health (e.g. Magruder et al., 2017). Although still often rejected, it is important also to publish research with negative findings, especially when it involves a replication study such as ‘Modification of episodic memories by novel learning: a failed replication study’ by van K. Van Schie et al. (2017).

## 4. EJPT metrics

### 4.1. Article downloads

#### 4.1.1. Article downloads by region in 2017

**Figure 4.**
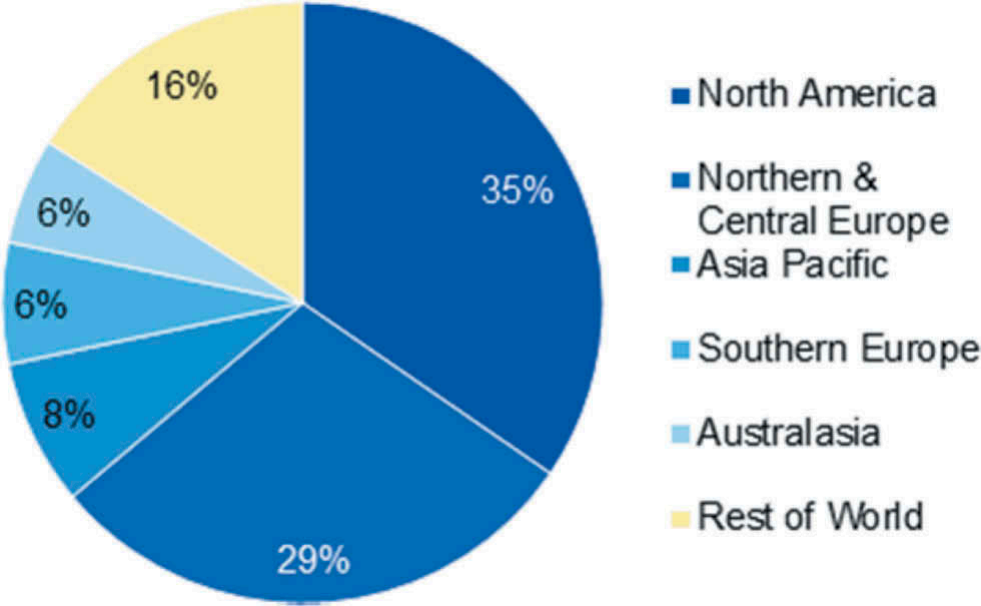
Downloads by continents in 2017.

Figure 4 shows the downloads per continent, a good distribution around the world. This Open Access policy encourages downloads from regions that are usually less reached by subscription journals behind paywalls.

#### 4.1.2. Top 3 downloads 2017

The most downloaded articles in 2017 were:

1. The neural correlates of childhood maltreatment and the ability to understand mental states of others (C. Van Schie et al., 2017);
2. A randomized controlled trial of brief Somatic Experiencing for chronic low back pain and comorbid PTSD symptoms (Elmose Andersen et al., 2014);
3. Six-year follow-up of the treatment of patients with dissociative disorders study (Myrick et al., 2017)

Interestingly, these three papers are probably in the top 3 most downloaded papers because of their clinical relevance.

### 4.2. Citations and impact factor

The journal’s 2016 Impact Factor is 3.278, increasing from 2.325 in 2015. EJPT ranks 20/121 in the Psychology, Clinical and 30/139 in Psychiatry JCR category.

The top 3 cited articles for this current impact factor are:

1. Resilience definitions, theory, and challenges: interdisciplinary perspectives (Southwick et al., 2014);
2. Psychotherapies for PTSD: what do they have in common? (Schnyder et al., 2015);
3. Evidence of symptom profiles consistent with PTSD and complex PTSD in different trauma samples (Elklit et al., 2014).

**Figure 5.**
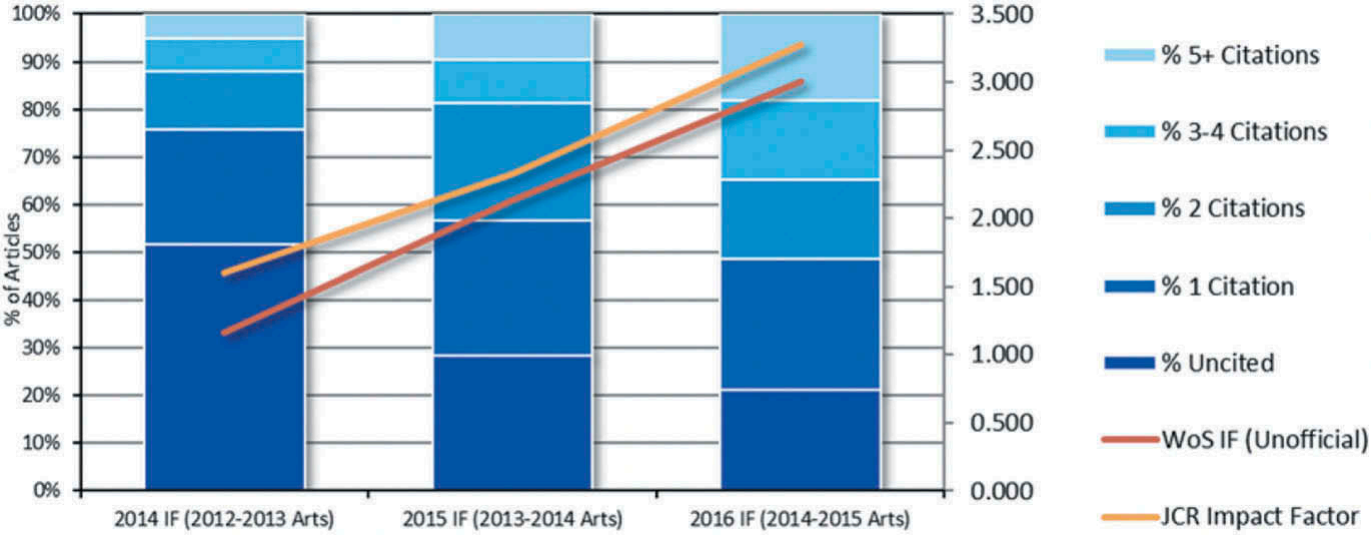
Article citations.

Figure 5 shows a positive trend that we expect to continue for the 2017 impact factor, and the welcome trend indicating that, over time, fewer papers have zero citations.

Our current acceptance rate is 68.4%, and the mean number of days until a first decision is 46 days. This includes some delays owing to the complex operation of the transfer of papers from the previous to the current publisher.

#### 4.2.1. For a specialist journal, why do we have such a good impact factor?

While having a wide scope (as long as trauma-related) and an open mind to any good research or clinical issues, and what is going on in society, we do publish a selected number of papers. Basically, an online journal has little space restrictions, but we do not aim to be a mega journal and will remain critical about our content. Our editorial team is doing an excellent job organizing the review process, allowing good research to be published, and they are helping authors to improve their work, especially those in emerging economies.

We are also very transparent about being Open Access. The proliferation of predatory journals resulting in zillions of invitations to publish our work in their journal seems hard to stop, even with advanced spam filters. EJPT would never invite authors to submit to the journal without being very clear about the APCs, whether fees are waived or not, and why we would invite someone (e.g. because they are keynote speakers at the ESTSS conference and we are creating a special issue based on the conference presentations).

Being an Open Access journal clearly helps readers to find and cite papers. At the same time, we should definitely not overstate the value of the impact factor; it is just one measure of impact. Altmetrics may actually better reflect important parameters as societal impact.

### 4.3. Altmetrics

Altmetrics have become more and more accepted measures of impact, for example in academic evaluations of societal impact of one’s work. Clicking on the altmetrics icon will show exactly what is said about the particular paper in news outlets, blogs, policy sources, tweeters, Facebook pages and even a Wikipedia page in this case. This information is present for all papers published in EJPT. Check it out!


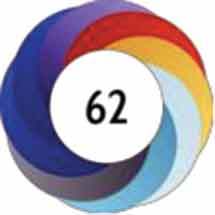
 In EJPT, the almetric score is highest for this paper: Distinguishing PTSD, Complex PTSD, and Borderline Personality Disorder: A latent class analysis (Cloitre et al., 2014).

### 4.4. Translations

With EJPT being read around the world, it is important to involve the readers as much as possible in their own language. Therefore, we are translating the abstracts and sometimes full papers in several languages (listed on www.estss.org). Since mid 2017, the Spanish and Chinese translations have been incorporated into the published article, including the pdf. Credits for this should go to Miriam Ramos and Yulan Qing, respectively, who have been volunteering to translate every abstract after acceptance within three working days.

ESTSS and the member societies are in the process of hosting the translations in their own language area (e.g. Dutch: www.ntvp.nl or German: www.degpt.de). All this involves an enormous amount of volunteer effort. Many thanks to all involved!

## 5. Moving forward: 2018

### 5.1. 2018: upcoming special issues

Again, in 2018, we aim to publish several special issues on important topics. Some are currently in their final stages (calls closed):

- Traumatic Loss *Guest Editors* Paul Boelen and Geert E. Smid;
- Children and Natural Disaster *Guest Editors*: Atle Dyregrov and William Yule;
- ISTSS Highlights 2016 *Guest Editor*: Paul Frewen;
- ESTSS Highlights 2017 – Child Maltreatment Across the Lifespan *Guest Editor*: Maj Hansen.

### 5.2. Special issues currently open for submissions

We currently have three open calls:

1. Complementary and Integrative Interventions for PTSD – *Guest Editors: Ariel Lang and Barbara Niles* Deadline 15 June 2018.

More and more people are seeking complementary, alternative or integrative ways to find help such as meditation, yoga, acupuncture, energy therapies and herbal supplements, for trauma-related problems. But what is the evidence for the efficacy and effectiveness of these treatments? What are the mechanisms of action?

(2) eHealth Applications in the Field of Traumatic Stress – *Guest Editors: Heleen Riper and Anne Bakker* Deadline: 15 May 2018.

Similarly, the development and use of eHealth including web-based and mobile apps (mHealth), online interventions, telepsychology, serious gaming and virtual reality are increasing rapidly. eHealth applications may be used for screening, monitoring, diagnostics, (early) self-help, prevention, treatment, relapse prevention and maintenance care, but despite the potential advantages and technological innovations, the empirical evidence for eHealth applications in the field of traumatic stress is still limited.

(3) Trauma and Trauma Sequelae in the Elderly – *Guest Editor: Brigitte Lueger-Schuester* Deadline: 15 April 2018.

Trauma and its sequelae in the elderly population are another rather under-researched topic. We still know very little about the long-term consequences of traumatization with respect to the assessment of disorders and their treatment, or about the effects of traumatization during older age.

Gender: We are preparing a call for papers for a special issue on Gender aspects in psychotraumatology.

Please see our journal homepage (www.tandfonline.com/ejpt) for details of the open calls on and the types of papers we are looking for.

### 5.3. Editorial team

We have had some changes in the (editorial team). Associate editor Chris Brewin who has been with us from the start, has retired, but we have found a strong team of new associate editors: Maj Hansen, University of Southern Denmark, Neil Roberts, Cardiff & Vale University Health Board and Cardiff University, U.K., and Siri Thoresen, Norwegian Centre for Violence and Traumatic Stress Studies (NKVTS) joined in 2017. I am very grateful to have this fantastic group of associate editors, but also guest editors, the editorial board and external reviewers that make this journal such a success. With the increasing number of publications, it becomes more and more difficult to find reviewers, but with a committed editorial board and external reviewers, we manage to provide high-quality reviews. In 2018, we will again try hard to meet the standards we set ourselves, and we hope we can count on you for your help. A journal is a truly collaborative effort!
